# Influence of Beverage Immersion and Repolishing on the Color Stability of CAD/CAM Restorative Materials: An In Vitro Study

**DOI:** 10.3390/ma19081519

**Published:** 2026-04-10

**Authors:** Umut Dağdeviren, Mine Betül Üçtaşlı, İrem Köklü Dağdeviren

**Affiliations:** 1 Department of Restorative Dentistry, Faculty of Dentistry, Gazi University, Ankara 06490, Türkiye; uctasli@gazi.edu.tr; 2 Department of Prosthodontics, Faculty of Dentistry, Gazi University, Ankara 06490, Türkiye; iremkoklu@gazi.edu.tr

**Keywords:** CAD/CAM, repolishing, color stability, color change, staining, resin nanoceramic, lithium disilicate, aesthetic properties

## Abstract

Long-term aesthetic success in dentistry largely depends on the color stability of restorative materials. This study investigated the color changes (ΔE_00_) of resin nanoceramic and lithium disilicate ceramic restorative materials used in computer-aided design and computer-aided manufacturing (CAD/CAM) systems following beverage immersion and after repolishing. One hundred specimens were prepared from lithium disilicate (Initial LiSi Block) and resin nanoceramic (Cerasmart), and polished. The specimens were divided into ten groups according to material and beverage type (*n* = 10) and immersed in distilled water, cola, tea, coffee, and turnip juice at 37 °C for 3 months. Color values were recorded at baseline, 1 week, 1 month, and 3 months, and after repolishing. ∆E_00_ values were calculated using the CIEDE2000 color difference formula. Data were analyzed using three-way repeated measures ANOVA and post hoc Tukey and Bonferroni tests (α = 0.05). Material type, beverage type, and immersion time significantly affected color stability (*p* < 0.05). The highest ∆E_00_ observed in the resin nanoceramic–tea group at 3 months (ΔE_00_ = 11.39 ± 1.76). Lithium disilicate demonstrated better color stability. After repolishing, all ΔE_00_ values were below the clinically acceptable limit (ΔE_00_ ≤ 1.8). Repolishing may help maintain the long-term aesthetic success of dental restorations in the oral environment.

## 1. Introduction

In recent years, the demand for aesthetic restorations that enhance patient satisfaction in dentistry has been gradually increasing. In this context, CAD/CAM technologies have facilitated the development of aesthetically successful restorations [1]. The terms ‘computer-aided design’ and ‘computer-aided manufacturing’ refer to the use of computer technologies in the design and fabrication processes of restorations [2]. This technology enables a fully digital workflow from design to fabrication, allowing the materials to exhibit superior mechanical properties and high precision. Furthermore, it optimizes clinical workflow by eliminating conventional laboratory steps and enabling indirect restorations to be fabricated chairside in a single session [3]. In these systems, restorative materials are typically supplied as prefabricated blocks, and restorations are fabricated from these blocks using a milling process [4]. The availability of a wide range of restorative materials used in chairside systems and their continuous development make it challenging for clinicians to select the most appropriate material [5]. Chairside CAD/CAM materials are classified into different categories, including glass ceramics, resin composites, hybrid ceramics, zirconia, and acrylic resins, and their mechanical, physical, and aesthetic properties should be carefully evaluated [6].

Lithium disilicate ceramics are among the most preferred glass ceramics for aesthetic restorations due to their superior aesthetic and mechanical properties. The manufacturing process involves two stages. First, the material is milled in a pre-crystallized form and then fired to achieve its final mechanical strength and aesthetic properties [7]. Recently, fully crystallized lithium disilicate ceramics that do not require firing and allow direct single-step clinical use have also been developed. The main advantage of these materials is their ability to reduce treatment time and overall cost [8].

Hybrid ceramics represent a relatively new group of chairside CAD/CAM materials that constitute an important development in the field of dental materials. These materials have been developed to combine the aesthetic properties of ceramics with the reduced brittleness and increased fracture resistance of composite resins [9]. They are classified into two different categories: polymer-infiltrated ceramic network and resin nanoceramic blocks [10]. Resin nanoceramics exhibit a dentin-like elastic modulus, which contributes to a more balanced distribution of occlusal stresses during mastication. In addition, they can be easily milled and repaired intraorally using resin composite materials [11]. Cerasmart is a resin nanoceramic material characterized by a resin matrix containing a high proportion of silica and barium glass nanoparticles, with approximately 71% inorganic filler content. This material is recommended for clinical use in various indirect restorative procedures, including inlays, onlays, veneers, and single-unit crown restorations [12]. Nevertheless, there are some concerns regarding the color stability of resin nanoceramics due to their resin content; this should be considered when selecting them for indirect restorations [13].

Maintaining color stability is equally critical to the clinical performance of a restoration as its mechanical durability. Over time, color changes occurring in the oral environment may adversely affect the aesthetic appearance of restorations [14]. Color changes in restorations are classified as intrinsic or extrinsic according to their underlying mechanisms. Intrinsic color changes result from physicochemical reactions within the deeper layers of the restoration and are often irreversible. In contrast, extrinsic color changes are caused by external factors such as pigment absorption, dietary habits, smoking, and plaque accumulation [15]. In addition, surface roughness plays an important role among the factors affecting color changes, as rough surfaces may increase the susceptibility of materials to staining, particularly from food and beverage pigments [16]. Previous studies [17,18] have reported that beverages with high pigment content, such as tea, coffee, and red wine, may adversely affect the color stability of restorative materials. Artificial aging protocols such as thermocycling and toothbrushing are also commonly used to evaluate the color stability of restorative materials [19,20]. Repolishing has been shown to effectively reduce short-term extrinsic color changes caused by beverages [21].

Although the color stability of restorative materials has been widely investigated using various aging and staining protocols, most existing studies [22,23,24] have focused on relatively short-term immersion periods, typically around 7–30 days of immersion, and data regarding color stability following long-term beverage exposure remain limited. Additionally, there are limited studies [21,25] evaluating the effectiveness of repolishing in reducing potential color changes. However, the long-term color stability and comparative performance of contemporary lithium disilicate ceramics and resin nanoceramic materials remain largely unknown. Therefore, this study aimed to investigate the influence of long-term (3-month) static immersion in five beverages (distilled water, coffee, cola, turnip juice, and tea) on the color stability of two CAD/CAM materials—a lithium disilicate ceramic and a resin nanoceramic—and to evaluate the effectiveness of repolishing in reducing color changes. In the present study, three null hypotheses were formulated and tested: (1) material type, beverage type, and immersion time do not significantly affect the color stability of the tested materials; (2) there is no interaction among material type, beverage type, and immersion time with respect to color change; and (3) the repolishing procedure applied after the 3-month immersion period does not significantly influence the color change in the tested materials.

## 2. Materials and Methods

### 2.1. Specimen Preparation

In this in vitro study, two different CAD/CAM materials were used: a resin nanoceramic (Cerasmart, GC, Tokyo, Japan) (CS) and a lithium disilicate ceramic (Initial LiSi Block, GC, Tokyo, Japan) (LS). Rectangular specimens measuring 12 mm × 14 mm and 2 mm thick were prepared from each CAD/CAM block with a low-speed diamond saw (IsoMet 4000, Buehler, Lake Bluff, IL, USA) under water cooling. A total of 100 specimens were prepared. The surface of each specimen was polished sequentially using silicon carbide abrasive papers (600, 1000, 2000, and 4000 grit) under running water at 300 rpm for 20 s per grit using a polishing machine (Mecapol P230, Presi, Grenoble, France). To ensure standardization, thickness measurements were obtained for each specimen using a digital micrometer (Mitutoyo Corp., Tokyo, Japan) capable of readings to the nearest 0.01 mm. Thereafter, baseline color measurements (T0) were conducted after storing the specimens in distilled water at 37 °C for 24 h. Information regarding the materials evaluated in this study and their compositions is summarized in Table 1.

### 2.2. Immersion Procedures

**Figure 1:**
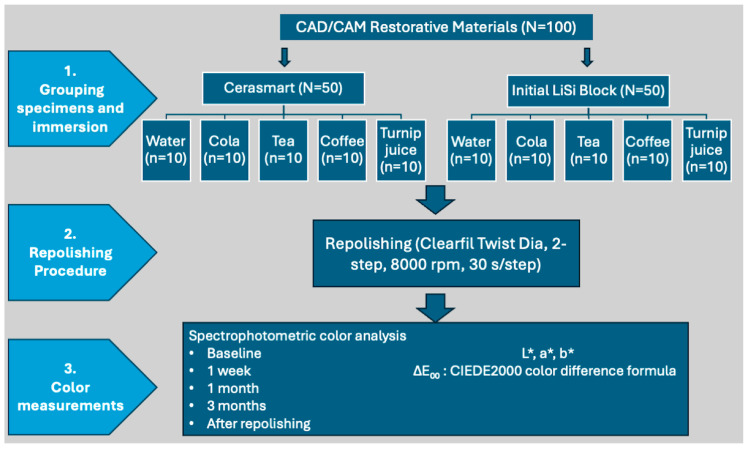
Flowchart of the study design.

The specimens were randomly divided into ten experimental groups based on the combination of two materials and five different beverages (*n* = 10 per subgroup). The beverages used in the study were distilled water (control group, pH of approximately 7.0), cola (Coca-Cola Company, Istanbul, Turkey; pH of approximately 2.5), coffee (Nescafe Classic, Nestlé, Turkey; pH of approximately 5.0), black tea (Yellow Label, Lipton, Istanbul, Turkey; pH of approximately 5.5), and turnip juice (Doğanay, Adana, Turkey; pH of approximately 3.5). Figure 1 illustrates the overall design and sequence of the study procedures.

Coffee and tea solutions were freshly prepared each time. The coffee solution was prepared by dissolving 3.6 g of coffee granules in 300 mL of boiling water with gentle stirring for 10 s, followed by filtration through filter paper. The tea solution was prepared by infusing one tea bag in 200 mL of boiling water for 5 min. Turnip juice and cola were used directly; a new bottle was used at each solution change.

All specimens were numbered from one to ten within each group to ensure standardization. For each specimen, 5 mL of the corresponding beverage was added into sealed containers using a syringe, and complete immersion of the specimens in the beverage was ensured. Except during color measurements, the specimens were incubated at 37 °C for 3 months. During the immersion procedure, color measurements were performed at three time points: 1 week (T1), 1 month (T2), and 3 months (T3). To minimize microbial proliferation and maintain the chemical stability of the beverages, the solutions were completely renewed every 48 h [26].

### 2.3. Repolishing Procedure

After the immersion procedure, all specimens were thoroughly cleaned with distilled water and carefully dried using paper towels. A standardized repolishing protocol was then carried out on the stained surfaces with a two-step polishing system (Clearfil Twist Dia, Kuraray, Tokyo, Japan). Medium- and fine-grit diamond-containing polishing wheels were sequentially used on the specimens for 30 s per wheel with a low-speed handpiece operating at 8000 rpm without water cooling. However, between each application step, the specimens were rinsed with water for 10 s and air-dried for 5 s. Following the repolishing procedure, color measurements were repeated to obtain the final values (T4).

### 2.4. Color Measurements

Color measurements were recorded with a dental spectrophotometer (Vita Easy Shade Advance 4.0, Vita Zahnfabrik, Bad Säckingen, Germany) at baseline (T0), after 1 week of immersion (T1), after 1 month (T2), after 3 months (T3), and following the repolishing procedure (T4). All measurements were performed by the same operator to reduce measurement variability. To standardize the measurement location, one surface of each specimen was marked with a diamond round bur, and measurements were taken from the unmarked opposite surface, with the central area targeting the same region at each time point. The device was calibrated prior to each measurement in line with the manufacturer’s instructions. Measurements were conducted under standardized lighting conditions using a D65 illuminant and a gray background. For each specimen, three consecutive readings were recorded, and the mean L*, a*, and b* values were recorded.

To account for baseline variations among the specimens, the color difference (ΔE_00_) for each sample at every time point was calculated relative to its own initial color coordinates (T0), and the CIEDE2000 color difference formula shown below was used for these calculations:

$$∆E_{00}=\sqrt{(\frac{∆L*}{K_{L}S_{L}}{)}^{2}+(\frac{∆C*}{K_{c}S_{C}}{)}^{2}+(\frac{∆H*}{K_{H}S_{H}}{)}^{2}+R_{T}(\frac{∆C*}{K_{c}S_{C}})\left(\frac{∆H*}{K_{H}S_{H}}\right)}$$

In this formula, ∆L*, ∆C* and ∆H* correspond to the differences in lightness, chroma, and hue between the compared specimens. The term R_T_ denotes a rotation component introduced to account for the combined influence of chroma and hue variations, particularly within the blue region of the color space. The coefficients S_L_, S_C_, and S_H_ serve as weighting factors that scale the overall color difference in relation to the L*, a*, and b* parameters. Moreover, K_L_, K_C_, and K_H_ are parametric adjustment constants incorporated to compensate for specific experimental conditions.

For this study, the parametric factors of the ΔE_00_ formula (K_L_, K_C_, and K_H_) were set to 1.0. According to ISO/TR 28642:2016 [27] and previously established threshold values in the literature, ΔE_00_ values ≤ 1.8 are considered the threshold of clinical acceptability, whereas ΔE_00_ values ≤ 0.8 are considered the perceptibility threshold [28].

### 2.5. Statistical Analysis

Statistical analysis of the data obtained in this study was performed using IBM SPSS Statistics 22 software (IBM Corporation, Armonk, NY, USA). The normality of the data distribution was assessed using the Kolmogorov–Smirnov and Shapiro–Wilk tests, and all data were found to follow a normal distribution. A three-way repeated measures ANOVA was used to evaluate the effects of material, beverage, and immersion time on the ΔE_00_ values. The assumption of sphericity for the repeated measures was assessed using Mauchly’s test. Because the sphericity assumption was violated, the degrees of freedom were adjusted using the Greenhouse–Geisser correction to prevent Type I error.

To determine specific group differences, the Tukey post hoc test was employed for the between-subjects factors (Material and Beverage) because it effectively controls the family-wise error rate across multiple pairwise comparisons without causing critical power loss. For the within-subjects factor (Immersion time), the Bonferroni correction was applied, as it provides robust and strict control for repeated measurements on the same specimens. Furthermore, to isolate and evaluate the specific effect of the repolishing procedure, a Paired Samples t-test was conducted to compare the ΔE_00_ values at the 3-month interval with the values obtained after repolishing for each material and beverage combination. For these comparisons, the mean difference, 95% confidence intervals (CI), and Cohen’s d effect sizes were reported. Partial eta squared (η_p_^2^) was used to report the effect sizes for the ANOVA model. All statistical analyses were performed at a significance level of *p* < 0.05.

A post hoc power analysis was conducted based on the observed effect size for the main factor “Material” (η_p_^2^ = 0.672). The corresponding Cohen’s f was calculated as 1.43, indicating a very large effect size. With the current sample size (*n* = 10 per subgroup) and α = 0.05, the statistical power was estimated to be greater than 0.99, suggesting that the study had adequate power to detect meaningful differences. 

## 3. Results

### 3.1. Overall Analysis of ΔE_00_

**Figure 2:**
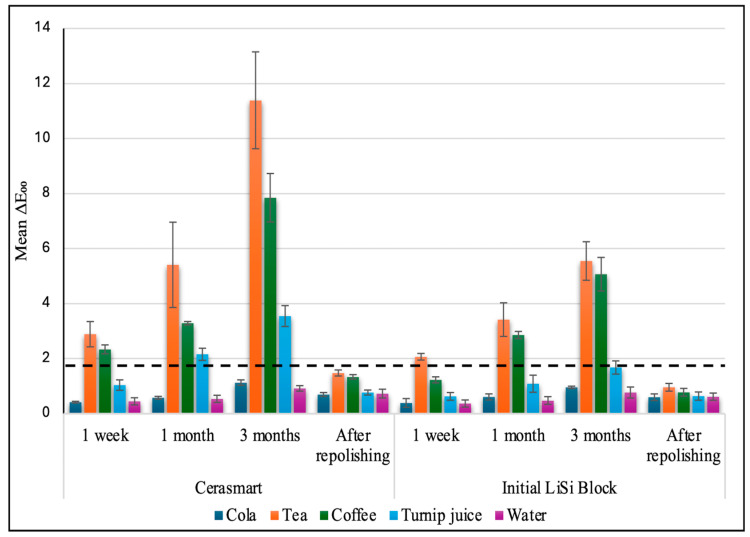
Mean ΔE_00_ values ± SD for tested materials. The dashed line indicates the clinical acceptability threshold (ΔE_00_ = 1.8).

The three-way repeated measures ANOVA (Table 2) revealed highly significant main effects for Material (F = 184.15, *p* < 0.001, $\eta_{p}^{2}$ = 0.672), Beverage (F = 472.31, *p* < 0.001, $\eta_{p}^{2}$ = 0.955), and Immersion time (F = 1165.49, *p* < 0.001, $\eta_{p}^{2}$ = 0.928). The large partial eta squared values indicate that all three factors had a profound clinical effect on color stability. Furthermore, all two-way and three-way interactions, including the Immersion time × Material × Beverage interaction (F = 36.41, *p* < 0.001, $\eta_{p}^{2}$ = 0.618), were statistically significant, indicating that the rate of staining over time was dependent on the specific combination of material and beverage. Figure 2 presents the mean ∆E_00_ values ± SD for the tested materials.

### 3.2. Effect of Beverage Type and Immersion Time on Color Stability

Regarding the effect of beverage type, all tested materials exhibited the highest ΔE_00_ values from the first week onward, following the general order: tea > coffee > turnip juice > cola ≈ distilled water (Table 3). Specifically, the Tukey post hoc test revealed no statistically significant difference between the cola and distilled water groups for either material at any time point (*p* > 0.05). Interestingly, at the 3-month interval, no statistically significant difference was found between the coffee and tea groups for the LS material (*p* = 0.119), whereas for the CS material, tea caused significantly greater staining than coffee (*p* < 0.001). For all beverages, ΔE_00_ values increased significantly as immersion time progressed from 1 week to 3 months (*p* < 0.001), demonstrating a strong main effect of immersion time (η_p_^2^ = 0.928).

### 3.3. Effect of Material Type on Color Stability

Statistically significant differences were found between the CS and LS materials at all time points during immersion in tea, coffee, and turnip juice beverages (*p* < 0.05). However, no significant difference was detected between the two materials in the cola and distilled water groups up to the 3-month interval (*p* > 0.05). The LS material demonstrated better color stability compared to CS.

### 3.4. Effect of Repolishing on Color Stability

The repolishing procedure significantly reduced the ΔE_00_ values across almost all material and beverage combinations after 3 months of immersion (*p* < 0.05). The exact mean differences, 95% CI, and Cohen’s d effect sizes are detailed in Table 4.

Paired samples t-tests revealed that repolishing provided the greatest reductions in ΔE_00_ values for specimens immersed in highly chromatogenic beverages (tea and coffee), exhibiting extremely large effect sizes (Cohen’s d ranging from 5.42 to 7.29) for both materials. For the CS groups, repolishing reduced the ΔE_00_ values of tea-immersed specimens by an average of 9.90 (*p* < 0.001) and coffee-immersed specimens by 6.50 (*p* < 0.001). Similarly, LS specimens showed a significant reduction of 4.59 in tea and 4.28 in coffee (*p* < 0.001). The only condition in which repolishing did not result in a statistically significant color correction was for the LS material immersed in distilled water (*p* = 0.080).

## 4. Discussion

This in vitro study evaluated the color stability of two CAD/CAM restorative materials with different structural compositions—a resin nanoceramic and a lithium disilicate ceramic—after immersion in commonly consumed beverages, as well as the effect of a repolishing procedure on color change performed following the immersion protocol. According to the findings, lithium disilicate ceramic demonstrated better color stability than the resin nanoceramic. Specimens immersed in coffee and tea exhibited the most pronounced and clinically unacceptable color changes, and these color changes significantly increased with immersion time. Therefore, the first null hypothesis, which stated that material type, beverage type, and immersion time would not significantly affect the color stability of the tested materials, was rejected. Moreover, significant interaction effects among material type, beverage type, and immersion time were observed for color change. Therefore, the second null hypothesis, which stated that no interaction would exist among these factors, was rejected. Furthermore, the repolishing procedure applied after immersion resulted in substantial and significant color recovery in all specimens and reduced the color change values to clinically acceptable levels; therefore, the third null hypothesis, which stated that the repolishing procedure would have no effect on the color change in the tested materials, was also rejected.

According to the literature, color changes occurring over time in dental restorations represent a major reason for aesthetic failure and restoration replacement [12,29]. A successful aesthetic restoration should first achieve complete color matching with the tooth and then maintain satisfactory color stability over the long term [30]. In order to simulate the long-term intraoral effects of beverages on restorations under accelerated laboratory conditions, a 3-month immersion period was applied. It has been reported in the literature that 24 h of continuous static immersion simulates approximately one month of clinical exposure [31]. This estimation is based on average beverage consumption patterns; accordingly, the time required to consume one cup of coffee is approximately 15 min, and the average daily coffee consumption is about 3.2 cups. Based on this assumption, a 24 h static immersion period corresponds to the effect of one month of beverage exposure. Therefore, the 3-month protocol was projected to represent approximately 7.5 years of cumulative chromogenic challenge. This duration was selected to approximate the 5–10-year clinical survival periods reported for CAD/CAM-fabricated indirect restorations, thereby enabling assessment of long-term color stability within a clinically meaningful framework [32].

The color changes in dental materials are commonly evaluated in the literature using CIE Lab or CIEDE2000 color difference formulas [33,34]. The CIEDE2000 formula provides results closer to human visual perception and offers higher perceptual uniformity, particularly when assessing small color differences [35]. For objective clinical evaluation, perceptibility and clinical acceptability thresholds are used. The clinical acceptability threshold is considered an important parameter for determining whether the observed color difference remains within aesthetically tolerable limits [36]. However, several studies in the literature have reported different visual threshold values. These variations reflect differences in evaluation conditions and should be taken into account when interpreting color difference results in a clinical context [37]. In this study, color changes were calculated according to the CIEDE2000 formula, and the resulting values were compared with the perceptibility and clinical acceptability thresholds of 0.8 and 1.8, respectively [28].

The results of this study indicate that LS, the fully crystallized lithium disilicate ceramic, exhibited better color stability than CS, the resin nanoceramic, across all beverage groups. This study confirms previous research findings that glass-ceramics are more resistant to staining agents compared to resin ceramics [38,39,40,41]. Resin-based restorative materials tend to exhibit relatively high surface free energy, which may contribute to increased susceptibility to staining and reduced color stability [42]. Furthermore, differences in staining between the two materials can also be attributed to variations in their microstructural composition, as the presence of resin increases the number of microvoids and filler–matrix interfaces in the organic phase, creating microstructural irregularities that facilitate the adhesion and penetration of pigments [25]. Since the beverages used in this study have polar characteristics, they can more easily interact with resin nanoceramic materials that contain an organic matrix [43]. In contrast, ceramic materials are primarily composed of crystalline minerals and a glassy matrix and have a denser microstructure, which, due to the absence of an organic resin matrix and their predominantly inorganic composition, interacts less with external factors such as water absorption and chemical diffusion, thereby making them more resistant to color changes [44]. While these results emphasize the impact of material composition on color changes, clinically unacceptable color differences were observed in both material groups for tea, coffee, and turnip juice after the 3-month immersion period. Therefore, although the selection of restorative material is important, the risk of staining under long-term exposure to pigmented beverages should be considered for both materials.

In this study, beverages commonly used in previous immersion tests [18,45,46], such as tea, coffee, and cola, were selected for the experimental groups, while distilled water served as the control. Additionally, turnip juice, a traditional fermented beverage produced by lactic acid fermentation and containing anthocyanin pigments, was included due to its potential to contribute to discoloration through pigment adsorption under acidic conditions, as well as the limited information available in the literature regarding this topic [47,48]. Previous studies [22,49] have reported that one-month immersion in coffee results in clinically unacceptable color changes in CS material. In the present study, both tea and coffee produced clinically unacceptable color changes in both material groups after one month of immersion. Dermanlı et al. [50] immersed CS material in tea and coffee for one week and observed significantly greater color changes in coffee compared to tea; however, in the current study, the CS-tea group exhibited significantly greater color changes than the CS-coffee group from the first week onward. This discrepancy may be attributed to the previously reported significant effects of different surface preparation protocols on color stability [51].

Koçak et al. [52] reported statistically significant color changes for the Cerasmart material in tea and coffee solutions after 7 and 30 days of immersion; however, these changes remained within clinically acceptable limits according to the CIELab formula. In contrast, the present study demonstrated clinically unacceptable color changes in the same solutions starting from the first week onward. This discrepancy may be attributed not only to methodological variables, such as specimen thickness and surface finishing procedures, but also to differences in the color difference formulas and clinical threshold values applied. Therefore, both methodological factors and clinical thresholds should be carefully considered when interpreting the significance of color changes.

In our study, as the immersion time increased, staining intensity also increased statistically; however, in the cola and distilled water groups, color changes remained below the clinical acceptability threshold even after 3 months. This suggests that the type of beverage may have a more decisive effect on staining than immersion time. Turnip juice, although causing higher color changes compared to cola and distilled water, resulted in less staining than tea and coffee.

Tea is rich in tannins, which can adsorb onto the material surface and penetrate the organic matrix, contributing to staining [53]. Coffee contains yellow pigments with different polarities; these pigments interact with the polymer matrix and are absorbed into the organic phase of resin-based materials, leading to staining [54]. Turnip juice contains anthocyanin pigments derived from purple carrots. These pigments have limited stability in aqueous solutions with low pH and may not adsorb sufficiently onto material surfaces. Additionally, the fermented nature of turnip juice may affect pigment stability. Anthocyanins have been reported to be sensitive to environmental factors such as light, temperature, and pH, and prone to degradation [55]. Therefore, the limited staining caused by turnip juice compared to tea and coffee can be attributed to the structural characteristics and low stability of its pigments.

Bagheri et al. [56] reported that the absence of yellow colored molecules in coffee may explain why cola causes less staining compared to coffee. This observation is consistent with our finding that cola exhibited color changes within clinical acceptability limits and suggests that the long-term consumption of pigmented beverages may play a significant role in staining. However, in the present study, statistically significant color changes were observed with increasing immersion time in distilled water despite the absence of pigment content. Similar findings have also been reported in previous studies [57,58], indicating that water sorption may affect the optical properties of restorative materials and lead to measurable color changes over time. Furthermore, in our study, the finding that the CS material exhibited greater color change than the LS material even during distilled water immersion appears to be consistent with literature reports suggesting that resin-containing restorative materials may be more susceptible to water sorption [59].

Staining of restorations can be effectively reduced through repolishing, depending on the type of material and the severity of staining [60]. Previous studies [21,25,45] have reported that repolishing, performed using polishing pastes or mechanical polishing methods on specimens exposed to various staining agents, significantly decreased staining. Similar observations have been reported in the literature. After a 3-month immersion period, repolishing was performed using a two-step mechanical polishing system, which markedly reduced staining in all experimental groups, with color changes remaining below the clinical acceptability threshold.

Long-term color stability is one of the fundamental properties that determine the clinical performance of aesthetic restorative materials, and unacceptable color changes are considered one of the primary reasons for the replacement of restorations [61]. The surface layer of restorations is generally the most susceptible region to extrinsic color change due to direct contact with staining agents [62]. In this regard, depending on the severity of staining, chairside repolishing may be considered a conservative maintenance strategy to restore color change to acceptable levels and reduce the need for restoration replacement due to aesthetic concerns.

This in vitro study has inherent limitations The specimens were prepared with flat surfaces and uniform thicknesses, unlike the morphological variability observed in clinical restorations. Although this approach facilitates standardized and reproducible color measurements, it may not fully reflect clinical conditions. This situation should be interpreted with caution, as the use of flat and homogeneous surfaces may alter staining behavior and the effectiveness of polishing procedures. During immersion, the specimens were exposed to the beverages on both surfaces, whereas in clinical conditions, only the outer surface of the restoration is in contact with beverages. This may have caused the observed color changes to be greater than those expected clinically. Additionally, oral dynamics such as tooth brushing, the oral microbiome, pH fluctuations, salivary buffering effects, intermittent exposure to beverages, and pellicle formation were not simulated in this study. Thermal aging that mimics intraoral temperature variations was not applied; this may limit the direct extrapolation of the findings to clinical conditions and may lead to overlooking the potential influence of factors such as toothbrushing simulation and thermocycling protocols on staining [20]. Furthermore, surface roughness evaluation before and after repolishing was not performed, which may limit the interpretation of the underlying mechanisms of color change, and the chemical characterization of the staining agents was not performed. Therefore, future studies are recommended to simulate more realistic intraoral conditions, incorporate thermal aging protocols, perform surface analysis using profilometry and scanning electron microscopy (SEM), include additional restorative materials, and employ anatomically contoured specimens that better represent clinical restorations. Nevertheless, the current results provide valuable insights into the long-term color stability of lithium disilicate ceramics and resin nanoceramics, highlighting the potential importance of repolishing in reducing staining.

## 5. Conclusions

Considering the limitations of this in vitro study, the following conclusions were reached:

1. Color changes were significantly influenced by the type of restorative material, the type of beverage, and the immersion time, as well as by the interactions among these factors.
2. The lithium disilicate ceramic (Initial LiSi Block) demonstrated superior color stability compared with the resin nanoceramic material (Cerasmart).
3. Beverages with high pigment content, particularly tea and coffee, exhibited the greatest staining potential and caused clinically unacceptable color changes in both materials from the first month of immersion.
4. Repolishing markedly reduced staining and restored color values to clinically acceptable levels.
5. In restorations where aesthetics is a primary concern, appropriate material selection and increasing patient awareness regarding dietary habits may be considered important factors. In addition, follow-up appointments including repolishing procedures may be considered as an approach that could contribute to reducing discoloration; however, the clinical effectiveness of such interventions should be interpreted with caution, given that the present findings are based on in vitro conditions and may not fully reflect clinical performance.

## Figures and Tables

**Table 1:** The materials and compositions used in the study.

Material	Composition	Brand	Manufacturer
Resin nanoceramic	Polymer phase: Bis-MEPP, UDMA, Polyfunctional methacrylate monomers (29 wt%) Ceramic phase: Silica and barium glass nanoparticles (Silica (20 nm), barium glass (300 nm)) (71 wt%)	Cerasmart	GC, Tokyo, Japan
Lithium disilicate	SiO_2_ (55–80 wt%), Li_2_O (10–30 wt%), other oxides (5–20 wt%)	Initial LiSi Block	GC, Tokyo, Japan

Bis-MEPP: Bis(p-methacryloxy(ethoxy)1–2 phenyl)propane, UDMA: Urethane Dimethacrylate, wt%: weight percentage.

**Table 2:** Three-way repeated measures ANOVA results for ∆E_00_.

Source of Variation	Type III Sum of Squares	df	Mean Square	F	*p*-Value	Partial Eta Squared ( $\eta_{p}^{2}$)
**Between-Subjects**						
Material	93.75	1	93.75	184.15	<0.001	0.672
Beverage	961.82	4	240.46	472.31	<0.001	0.955
Material * Beverage	80.83	4	20.21	39.69	<0.001	0.638
Error (Between)	45.82	90	0.51			
**Within-Subjects**						
Immersion time	381.37	2	190.69	1165.49	<0.001	0.928
Immersion time * Material	41.40	2	20.70	126.52	<0.001	0.584
Immersion time * Beverage	263.19	8	32.90	201.08	<0.001	0.899
Immersion time * Material * Beverage	47.66	8	5.96	36.41	<0.001	0.618
Error (Within)	29.45	180	0.16			

Three-way repeated-measures ANOVA Test * *p* < 0.05., Mauchly’s test indicated that the assumption of sphericity had been violated; therefore, degrees of freedom (df) and mean squares for within-subjects factors were adjusted using the Greenhouse-Geisser correction (ε = 0.538). Bold text indicates the main sources of variation (Between-Subjects and Within-Subjects) in the ANOVA model.

**Table 3:** Mean ΔE_00_ values, SDs, and statistical significance levels at different time points for the tested materials.

Material	Beverage	1 Week Mean ± SD [95% CI]	1 Month Mean ± SD [95% CI]	3 Months Mean ± SD [95% CI]	After Repolishing Mean ± SD [95% CI]
CS	Distilled Water	0.45 ± 0.13 [0.37–0.53] ^A^	0.54 ± 0.13 [0.46–0.62] ^A^	0.92 ± 0.10 [0.86–0.98] ^A^	0.73 ± 0.16 [0.63–0.83] ^A^
	Cola	0.42 ± 0.03 [0.40–0.43] ^A^	0.58 ± 0.05 [0.55–0.62] ^A^	1.13 ± 0.10 [1.07–1.19] ^A^	0.70 ± 0.07 [0.66–0.74] ^A^
	Tea	2.89 ± 0.46 [2.60–3.18] ^D^	5.41 ± 1.55 [4.45–6.37] ^D^	11.39 ± 1.76 [10.30–12.48] ^D^	1.48 ± 0.11 [1.41–1.55] ^C^
	Coffee	2.34 ± 0.16 [2.25–2.44] ^C^	3.29 ± 0.06 [3.25–3.33] ^C^	7.85 ± 0.88 [7.30–8.39] ^C^	1.34 ± 0.08 [1.29–1.39] ^B^
	Turnip Juice	1.04 ± 0.19 [0.92–1.16] ^B^	2.16 ± 0.22 [2.02–2.29] ^B^	3.55 ± 0.38 [3.31–3.78] ^B^	0.77 ± 0.09 [0.72–0.83] ^A^
LS	Distilled Water	0.37 ± 0.13 [0.29–0.45] ^A^	0.48 ± 0.14 [0.39–0.57] ^A^	0.77 ± 0.20 [0.65–0.90] ^A^	0.62 ± 0.13 [0.54–0.70] ^A,B^
	Cola	0.39 ± 0.16 [0.30–0.49] ^A^	0.61 ± 0.11 [0.54–0.68] ^A^	0.96 ± 0.04 [0.93–0.98] ^A^	0.60 ± 0.12 [0.53–0.68] ^A^
	Tea	2.07 ± 0.12 [2.00–2.15] ^D^	3.42 ± 0.61 [3.04–3.80] ^D^	5.55 ± 0.70 [5.12–5.99] ^C^	0.96 ± 0.14 [0.87–1.04] ^C^
	Coffee	1.23 ± 0.11 [1.16–1.30] ^C^	2.86 ± 0.13 [2.77–2.94] ^C^	5.07 ± 0.61 [4.69–5.45] ^C^	0.78 ± 0.14 [0.70–0.87] ^B^
	Turnip Juice	0.63 ± 0.14 [0.55–0.71] ^B^	1.09 ± 0.31 [0.90–1.28] ^B^	1.68 ± 0.24 [1.54–1.83] ^B^	0.64 ± 0.15 [0.55–0.74] ^A,B^

CS: Cerasmart; LS: Initial LiSi Block. Three-way repeated-measures ANOVA and Tukey post hoc test (*p* < 0.05). Different uppercase letters within columns indicate statistically significant differences between beverage groups.

**Table 4:** Paired Samples *t*-test results comparing ΔE_00_ values at 3 months versus after repolishing.

Material	Beverage	Mean Difference (ΔE_00_)	95% CI for Difference	t-Value	*p*-Value	Effect Size (Cohen’s d)
CS	Distilled water	0.19	[0.05, 0.33]	3.15	0.012	0.99 (Large)
	Cola	0.43	[0.35, 0.51]	12.60	<0.001	3.98 (Large)
	Tea	9.91	[8.60, 11.21]	17.14	<0.001	5.42 (Large)
	Coffee	6.51	[5.84, 7.17]	22.07	<0.001	6.98 (Large)
	Turnip juice	2.77	[2.47, 3.08]	20.51	<0.001	6.49 (Large)
LS	Distilled water	0.15	[−0.02, 0.33]	1.97	0.080	0.62 (Medium)
	Cola	0.35	[0.25, 0.46]	7.91	<0.001	2.50 (Large)
	Tea	4.59	[4.08, 5.11]	20.11	<0.001	6.36 (Large)
	Coffee	4.28	[3.86, 4.70]	23.07	<0.001	7.29 (Large)
	Turnip juice	1.04	[0.85, 1.23]	12.41	<0.001	3.92 (Large)

Mean Difference represents [3 Months ΔE_00_]–[After Repolishing ΔE_00_] A positive value indicates a reduction in staining after repolishing. *p* < 0.05 indicates statistical significance.

## Data Availability

The original contributions presented in this study are included in the article. Further inquiries can be directed to the corresponding author.

## Abbreviations

The following abbreviations are used in this manuscript:

CAD/CAM	Computer-Aided Design/Computer-Aided Manufacturing
ISO/TR	International Organization for Standardization/Technical Report
∆E_00_	CIEDE2000 color difference
